# Commentary: Concurrent Imitative Movement During Action Observation Facilitates Accuracy of Outcome Prediction in Less-Skilled Performers

**DOI:** 10.3389/fpsyg.2018.02561

**Published:** 2018-12-18

**Authors:** Wacław Petryński

**Affiliations:** Katowice School of Economics, Katowice, Poland

**Keywords:** Bernstein's theory, modalities' ladder, motor scenario, motor habit, tactics

In the paper “Concurrent Imitative Movement during Action Observation Facilitates Accuracy of Outcome Prediction in Less-Skilled Performers” Unenaka et al. (2018) have presented the comparison of experimental results obtained in groups of skilled and less skilled basketball players.

Their results remain in good compatibility with the conceptual models of modalities' ladder (Petrynski, 2014, 2016, p. 60; Petrynski, 2018) and movements' management matrix (Petrynski, 2016, p. 133). Both they are based on the theory of Bernstein (1947, 1996).

Let us term the “motor operation” an intentionally organized set of movements, aimed at solving of a specific task in environment (Petrynski, 2016, p. 14). While taking as a base the Bernstein's theory, it is possible to invent the modalities' ladder, i.e., a hierarchical system of information processing modalities, internal motor operation patterns, particular classes of motor operations, and movements' control modes as presented in Table 1.

**Table 1:** The modalities' ladder.

**Bernstein's level**	**Information processing modality**	**Internal motor operation pattern**	**Class of a motor operation**	**Movements' control mode**
E	Symbolic	No real motor operation pattern	No real motor operation	Politics
D	Verbal	Program	Performance	Strategy
C	Teleceptive, mainly visual	Scenario	Habit	Tactics, “measure-in-eye”
B	Contactceptive, mainly haptic	Template	Automatism	Technique, movements' harmony
A	Proprioceptive	Coupling	Reflex	Strength control, “feeling-in-hand”

Politics means adjusting the external conditions to the planned (usually not realizable here and now) performance rather, and not embedding any realizable performance in physically existing environment.

The researches presented by Unenaka et al. concern the C-level (Petrynski, 2016, p. 26; Petrynski, 2018). At that level, the mechanism of imitation is supported by activity of mirror neurons Rizzolatti, 2005. They make an innate system (i.e., being a part of a biological “hardware” rather, and not “software”), supporting the process of imitation, being an important learning mechanism in animals and humans.

Creation of the internal motor operation patterns is aimed at reducing the “intellectual costs” and thus enhancing the efficiency of a motor operation. While not yet fully shaped, such a pattern needs feedback control mode, typical for learning process. It is time-consuming and “expensive” in terms of mental effort, because it needs identification of physical stimuli in environment, associating them with the just being performed operation and creation of possible corrections. In short, it needs engagement of precious attention. Such a mechanism is typical for less-skilled performers, e.g., basketball players.

When a specific motor operation pattern (at C-level—scenario; Petrynski, 2016, p. 52; Petrynski, 2018) is already fully shaped, the intellectual cost decreases, and efficiency increases. A skilled basketball player disposes of reliable scenarios, ready to immediate use, hence s/he does not need any more to detect and identify the extrinsic stimuli (i.e., to engage attention), like in the process of learning. S/he switches to swift feedforward control mode and does not need any more a current (or concurrent) extrinsic informational support.

In the movements' management matrix model there is no dichotomous division: feedback (attention on)—feedforward (attention off). Somewhere in the midway there is a “stand by” state of attention—less “expensive” than “full feedback,” but not as “cheap” (in terms of attention engagement) as “full feedforward.” Hence, in the movements' management matrix the feedback and feedforward control modes make a continuous space, with attention fully active, more or less “sleeping,” and completely inactive.

If the feedforward (intrinsic control) becomes stronger than the influence of feedback (extrinsic stimuli), then the mental self-confidence may not overlap with a genuine reliability. Intentional provoking of such a false self-confidence makes an essence of, e.g., faking in sport.

Both the control modes—feedback, typical for less-skilled performers, and feedforward, used by skilled ones—may be also joined with the crystallized and fluid intelligence, respectively, as by R. Cattell (Gerrig, 2013, p. 248).

It seems instructive to conclude this comment with the remark of eighteenth century historian E. Gibbon, who stated, “*the power of instruction is seldom of much efficacy, except in those happy dispositions where it is almost superfluous”* (Gibbon, 1995, p. 79). Such an “informational frugality” makes also the essence of Bernstein's degrees of freedom reduction rule (Bernstein, 1947, p. 20), which enables transformation of non-controllable movements' creation systems into controllable ones. While perceived dynamically, it may be associated with the most advanced quality of attention, i.e., its switchability. It is also congruent with the “007 Principle” by A. Clark: “…to know only as much as you need to know to get the job done.” (Clark, 1993, p. 64).

## Author Contributions

The author confirms being the sole contributor of this work and has approved it for publication.

### Conflict of Interest Statement

The author declares that the research was conducted in the absence of any commercial or financial relationships that could be construed as a potential conflict of interest.
